# Apoptotic cell therapy for cytokine storm associated with acute severe sepsis

**DOI:** 10.1038/s41419-020-02748-8

**Published:** 2020-07-15

**Authors:** Netanel Karbian, Avraham Abutbul, Raja el-Amore, Ran Eliaz, Ronen Beeri, Barak Reicher, Dror Mevorach

**Affiliations:** 1 Rheumatology and Rare Disease Research Center, The Wohl Institute for Translational Medicine, Hadassah-Hebrew University Medical Center and School, Jerusalem, Israel; 2 Intensive Care Unit, Hadassah-Hebrew University Medical Center, Jerusalem, Israel; 3 Department of Cardiology, Hadassah-Hebrew University Medical Center, Jerusalem, Israel; 4 Enlivex Therapeutics Ltd., Ness-Ziona, Israel; 5 Department of Medicine, Hadassah-Hebrew University Medical Center, Jerusalem, Israel

**Keywords:** Inflammatory diseases, Cell death and immune response, Sepsis, Translational immunology, Experimental models of disease

## Abstract

Sepsis has no proven pharmacologic treatment other than appropriate antibiotic agents, fluids, vasopressors as needed, and possibly corticosteroids. It is generally initiated mainly by the simultaneous recognition by various components of the innate immune system of either pathogen-associated molecular patterns (PAMPs) or damage-associated molecular patterns (DAMPs). In the current study, we employed the murine cecal ligation and puncture (CLP) model for sepsis to evaluate the effect of post-CLP infusion of apoptotic cells (Allocetra-OTS) on a CLP severe sepsis model. Cardiovascular evaluation, acute kidney injury (AKI), acute liver injury (ALI), and hematological and metabolic function were evaluated. Cytokine and chemokine profiles were measured by Multiplex ELISA and mitochondrial function, and glycolysis by Seahorse. The Murine Sepsis Score (MSS) was used for disease severity definition. CLP mice had low blood pressure, poor cardiac output, and lung dysfunction, as well as AKI, ALI, and thrombocytopenia, which correlated with the MSS and corresponded to a cytokine/chemokine storm. Apoptotic cell administration markedly improved the cytokine and chemokine storm and restored the impaired mitochondrial and glycolytic function in white blood cells leading to increased survival, from 6 to 60% (*P* < 0.0001), together with a significant improvement in organ dysfunction. We conclude that the deleterious immune response in CLP-induced sepsis can be successfully modified by apoptotic cell infusion.

## Introduction

Sepsis, which has been identified by the World Health Organization (WHO) as a global health priority, has no proven pharmacologic treatment other than appropriate antibiotic agents, fluids, vasopressors as needed, and possibly corticosteroids^1–4^. Reported death rates among hospitalized patients range between 30 and 45%^5–10^.

Sepsis is generally initiated by simultaneous recognition of either pathogen-associated molecular patterns (PAMPs) or damage-associated molecular patterns (DAMPs), by components of the innate immune system^11,12^. Recognition induces a complex intracellular signaling system with redundant and complementary activities, and activation of these multiple signaling pathways ultimately leads to the expression of several common classes of genes that are involved in inflammation, adaptive immunity, and cellular metabolism^13^.

Sepsis elicits dysregulated immune responses manifested by both a cytokine/chemokine elevation (also known as ‘cytokine storm’) and immune suppression, which correlates well with increased disease severity and poor prognosis^11,12,14–16^. This unbalanced immune response deleteriously affects physiological homeostasis of vital organs, including the kidney, liver, lungs, and heart, and often evolves into multi-organ failure, also termed multiple organ dysfunction syndrome (MODS)^17,18^.

Billions of cells undergo apoptosis every hour in the human body. Apoptotic cells themselves are major contributors to the “non-inflammatory” nature of the engulfment process, some by secreting thrombospondin-1 (TSP-1) or adenosine monophosphate and possibly other immune-modulating “calm-down” signals that interact with macrophages and dendritic cells (DCs). Apoptotic cells also produce “find me” and “tolerate me” signals to attract and immunomodulate macrophages and DCs that express specific receptors for these signals^19^.

The pro-homeostatic nature of apoptotic cell interaction with the immune system is illustrated in known apoptotic cell signaling events in macrophages and DCs that are related to toll-like receptors (TLRs), nuclear factor κB (NF-κB), inflammasome, lipid-activated nuclear receptors, Tyro3, Axl, and Mertk receptors. In addition, induction of signal transducers, activation of transcription, and suppression of cytokine signaling lead to a pro-homeostatic immune system effect following immune response, with downregulation of both anti- and pro-inflammatory cytokines derived from PAMPs and DAMPs, in both animals and in vitro models^20,21^.

In the current study, we employed the murine cecal ligation and puncture (CLP) model. This model is proposed to more closely replicate the nature and course of human clinical sepsis than other models and is considered by many researchers as the gold-standard animal model of reproducible sepsis^22–24^. We used this model to evaluate the effect of donor apoptotic cells, which were shown to have a rebalancing effect on the immune system^20,25^ when administered in combination with fluid resuscitation and antibiotic treatment. This report summarizes the effect of apoptotic cells administered 4 h after the end of CLP on the development of CLP-induced sepsis in female C57BL/6 mice.

## Results

### Evaluation of the Murine Sepsis Score (MSS) clinical scoring system as a surrogate indicator for organ dysfunction in CLP mice

A MODS-like disease has been previously reported in murine CLP models^26–29^; however, histopathological analysis of organ dysfunction may not be an effective research tool for the development of therapeutic approaches in this model because it is a terminal procedure, requiring a large number of mice. In addition, histopathological results often show no differences between experimental groups and fail to correlate with disease severity and outcomes^30–32^. Thus, finding diagnostic tests for organ dysfunction in septic mice that strongly correlate with the MSS may be a clinically relevant research tool for sepsis.

Twenty-four hours post-CLP, each mouse (fluid- and ertapenem-treated, *N* = 40) was assigned a clinical score and weighed, and blood samples were taken for further analysis. Mice were sacrificed and their lungs were harvested and weighed. To elucidate the effects of CLP on organ dysfunction and its correlation with the MSS, blood was tested for multiple parameters of organ dysfunction relating to five major systems: cardiovascular, respiratory, renal, hepatic, and hematological, as well as complement and several metabolites (Table 1).

**Table 1:** Organ dysfunction analysis 24 h post CLP.

System	Parameter	Median of naive [IQR]		Median of CLP + ertapenem [IQR]		^b^*P*- Value	^c^Correlation to clinical score	^d^AUC of ROC curve
Respiratory	venous pCO_2_ (mmHg)	44.75 [42.0, 59.28];	*N* = 8	53.2 [43.4, 79.8];	*N* = 11	n.s.	No	N/A
	venous pO_2_ (mmHg)	46.45 [39.15, 59.98];	*N* = 8	57.4 [51.1, 63.3];	*N* = 13	n.s.	0.5934	N/A
	^a^pH	7.3245 [7.31, 7.341];	*N* = 9	7.1995 [7.02, 7.274];	*N* = 12	0.0089	−0.7792	0.8438
	^a^Lung/body weight (w/w)	0.0069 [0.0065, 0.007];	*N* = 21	0.0079 [0.0071, 0.0089];	*N* = 40	<0.0001	0.743	0.8494
Renal	Creatinine (mg/dL)	0.17 [0.145, 0.205];	*N* = 9	0.16 [0.11, 0.31];	*N* = 15	n.s.	No	N/A
	^a^Urea (mg/dL)	37.1 [33.9, 42.5];	*N* = 9	116.6 [67.8, 789.1];	*N* = 15	<0.0001	0.8852	1
	Cystatin C (ng/ml)	650 [600, 700];	*N* = 4	750 [525, 1575];	*N* = 16	n.s.	0.6196	N/A
	^a^NGAL (ng/ml)	150 [100, 275];	*N* = 4	35850 [23350, 50975];	*N* = 16	0.0004	0.7572	1
Hepatic	^a^Total protein (g/dL)	5.54 [5.405, 5.61];	*N* = 9	4.14 [3.77, 4.32];	*N* = 15	<0.0001	−0.865	1
	^a^Albumin (g/dL)	4.2 [4.05, 4.3];	*N* = 9	2.9 [2.6, 3.1];	*N* = 15	<0.0001	−0.8333	1
	Globulin (g/dL)	1.33 [1.235, 1.41];	*N* = 9	1.26 [1.12, 1.36];	*N* = 15	n.s.	−0.5312	N/A
	^a^AST (U/L)	345 [290, 519];	*N* = 9	1003 [873, 1328];	*N* = 15	<0.0001	0.7268	0.9852
	^a^ALT (U/L)	133 [94.5, 197.5];	*N* = 9	374 [327, 502];	*N* = 15	<0.0001	0.8216	0.9926
	^a^Alkaline Phosphatase (U/L)	192 [169, 202];	*N* = 9	101 [93, 110];	*N* = 15	<0.0001	−0.8432	1
	Total Bilirubin (mg/dL)	0.09 [0.065, 0.1];	*N* = 9	0.09 [0.07, 0.12];	*N* = 15	n.s.	No	N/A
Hematopoietic	^a^WBC (10^3^/µL)	2.585 [2.19, 3.605];	*N* = 8	1.94 [1.06, 2.18];	*N* = 15	0.0017	No	0.8833
	RBC (10^6^/µL)	9.935 [8.28, 10.17];	*N* = 8	8.36 [8.12, 9];	*N* = 15	n.s.	No	N/A
	Hemoglobin (g/dL)	14.85 [12.48, 15];	*N* = 8	12.4 [12, 14];	*N* = 15	n.s.	No	N/A
	HCT (%)	46.4 [41.55, 47.7];	*N* = 8	40.9 [39.3, 43.1];	*N* = 15	n.s.	No	N/A
	MCV (fL)	47.7 [46.25, 50.45];	*N* = 8	47.9 [46.3, 49.4];	*N* = 15	n.s.	No	N/A
	MCH (pg)	14.95 [14.75, 15.1];	*N* = 8	14.9 [14.7, 15.3];	*N* = 15	n.s.	No	N/A
	MCHC (g/dL)	31.7 [30.23, 32.28];	*N* = 8	31.2 [30.5, 33.3];	*N* = 15	n.s.	No	N/A
	^a^Platelets (10^3^/µL)	642.5 [548.5, 812.8];	*N* = 8	99 [87.25, 228.5];	*N* = 14	<0.0001	−0.7099	1
	Neutrophils (10^3^/µl)	0.51 [0.3175, 0.8125];	*N* = 8	0.31 [0.17, 0.39];	*N* = 15	0.0382	−0.4531	N/A
	Lymphocytes (10^3^/µl)	1.965 [1.723, 3.175];	*N* = 8	1.55 [0.68, 1.9];	*N* = 15	0.0275	No	N/A
	Monocytes (10^3^/µl)	0 [0, 0];	*N* = 8	0 [0, 0.04];	*N* = 15	N/A	No	N/A
	Eosinophils (10^3^/µl)	0 [0, 0];	*N* = 8	0 [0, 0];	*N* = 15	N/A	N/A	N/A
	Basophils (10^3^/µl)	0 [0, 0];	*N* = 8	0 [0, 0];	*N* = 15	N/A	N/A	N/A
Complement	^a^C3a (ng/ml)	8903 [7769, 11426];	*N* = 4	4835 [4216, 5652];	*N* = 16	0.0029	−0.7183	0.9531
	C5a (ng/ml)	1102 [992, 1664];	*N* = 4	1809 [1631, 2492];	*N* = 16	0.0219	No	0.875
Metabolites	Cholesterol (mg/dL)	97 [92.5, 100];	*N* = 9	108 [88, 140];	*N* = 15	n.s.	No	N/A
	Glucose (mg/dL)	151 [118.5, 178];	*N* = 17	59.5 [42.75, 122.3];	*N* = 28	0.0001	−0.3227	0.8288
	Lactate (mg/dL)	19.5 [17.25, 24.75];	*N* = 8	13 [9.5, 20];	*N* = 13	0.0255	No	N/A
Electrolytes	Phosphorus (mg/dL)	8.8 [8.3, 9.1];	*N* = 9	10.2 [8.5, 13.7];	*N* = 15	n.s.	0.6705	N/A
	Sodium (mmol/L)	151 [147.8, 153];	*N* = 17	154.1 [150.1, 158];	*N* = 26	0.0125	No	N/A
	Potassium (mmol/L)	5.7 [5.225, 5.9];	*N* = 17	6.6 [6.1, 7.3];	*N* = 27	0.0001	0.4993	0.8279
	Chloride (mmol/L)	115.6 [113.5, 117];	*N* = 17	121.1 [119, 125.3];	*N* = 27	<0.0001	0.5063	0.939

^a^Significant difference between CLP mice and naive mice with a strong correlation to MSS Clinical score (−0.7 > ρ-Spearman > 0.7).

^b^Mann–Whitney two-tailed nonparametric *t*-test.

^c^ρ-Spearman.

^d^Naive versus CLP mice.

### Severe cardiovascular and respiratory dysfunction in CLP mice

**Fig. 1:**
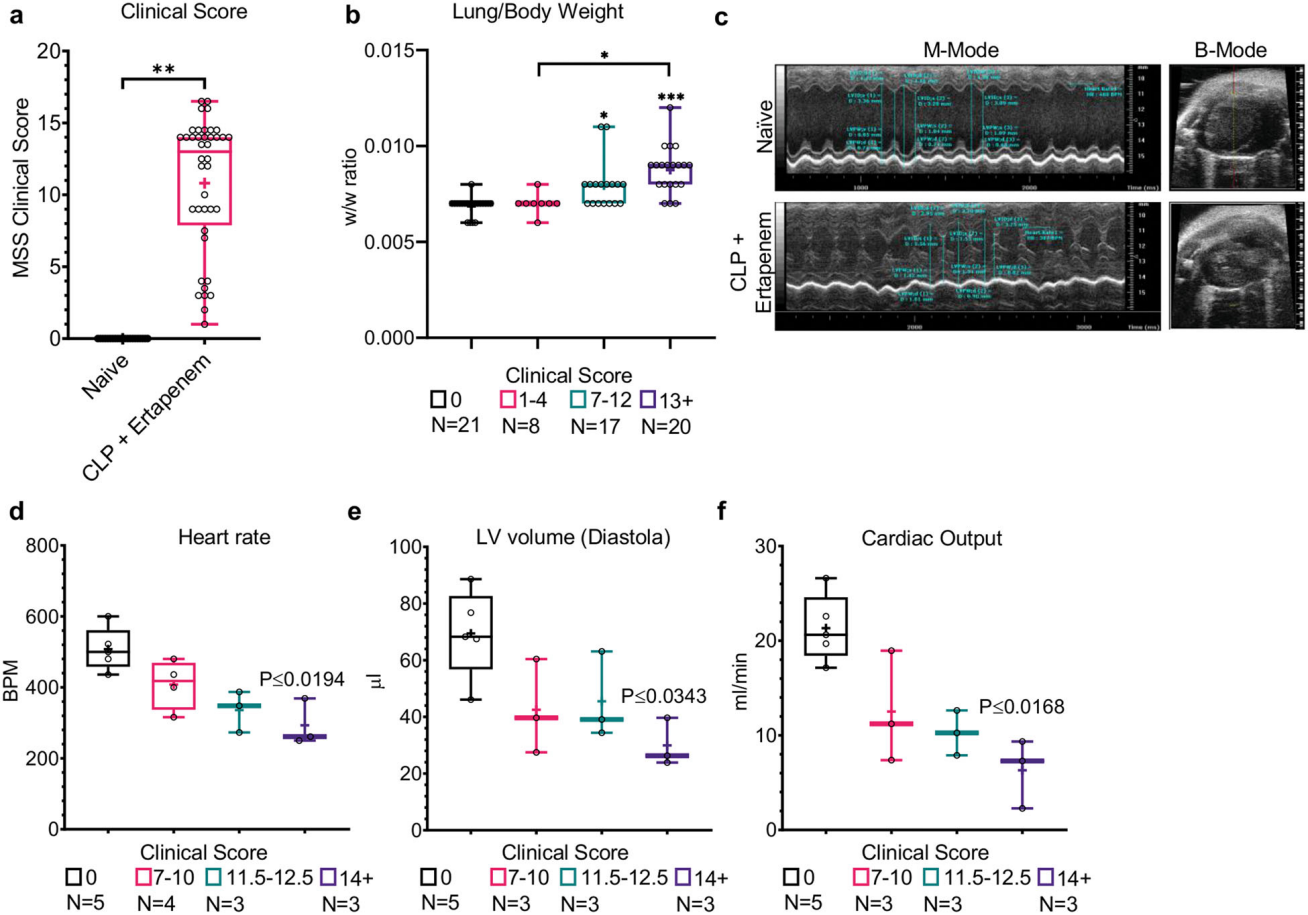
CLP mice display signs of respiratory and cardiovascular dysfunction that correlate with sepsis severity. **a** Twenty-four hours post-CLP, naive mice (*n* = 21) showed no signs of illness, while the majority of CLP mice (*n* = 40) had severe clinical signs (median MSS of 13; 95% CI of the median 9–14); **P < 0.0001 by a two-tailed Mann–Whitney test. **b** The lung-to-body weight ratio significantly increases with sepsis severity. **c** Representative 2D echocardiograms of naive (top panels) and CLP mice (bottom panels), showing the time-lapse view (M-Mode) and top view (B-Mode). LV internal distances, heart rate, and posterior wall thickness were measured for the calculation of various parameters of LV structure and function, including **d** heart rate, **e** LV volume, and **f** cardiac output. Data are presented as the median within the inter-quartile range (IQR); mean values are marked with a ‘+’ sign; error bars represent the minimum and maximum; group sizes (*N*) are indicated below their respective legends; *P ≤ 0.01, **P ≤ 0.001, ***P ≤ 0.0001 by the Kruskal–Wallis nonparametric ANOVA, with multiple-comparisons adjusted by using the Dunn’s test. *P* values above the bars indicate the significant differences from the control group, and those above the brackets indicate the significant differences between the two other groups.

The cardiovascular system is among the first to be affected in mice with CLP-induced sepsis^27,28,33^. Accordingly, our attempts at noninvasive measurement of murine blood pressure were not successful because the systolic blood pressure was below the instrument’s detection limit of <50 mmHg, further emphasizing the severity of sepsis. Lung dysfunction was evident by increased lung weight (normalized to body weight) due to fluid retention implicating heart failure and/or acute respiratory distress syndrome. The increase in lung weight strongly correlated with the MSS (Table 1; ρ-Spearman = 0.743), and accordingly significance was greater in mice with severe than those with moderate-severe sepsis (Fig. 1b; *P* ≤ 0.01 and *P* ≤ 0.0001 for MSS of 7–12 and 13+, respectively). Though CLP mice had no apparent structural myocardial damage (Fig. 1c, top view; B-Mode) they had a significantly lower heart rate (Fig. 1c, M-Mode) than naive mice, with a strong inverse correlation to the MSS (Supplementary Table 1; ρ-Spearman = −0.878), and the reduction was most significant in mice with severe sepsis (Fig. 1d; *P* ≤ 0.0194). Although the fractional shortening (FS) and ejection fraction (EF) were not significantly different between the groups (Supplementary Table 1; *P* = n.s.), CLP mice had significantly lower diastolic left ventricular (LV) volume with a strong inverse correlation to clinical score (Supplementary Table 1; ρ-Spearman = −0.701), and severely septic mice had the lowest LV volume (Fig. 1e; *P* ≤ 0.0343). The systolic LV volume and the measured LV area were also significantly lower in septic mice (Supplementary Table 1). Accordingly, cardiac output of CLP mice was severely impaired and, again, strongly and inversely correlated with disease severity (Supplementary Table 1; ρ-Spearman = −0.799 and Fig. 1f).

### Acute kidney injury (AKI)

**Fig. 2:**
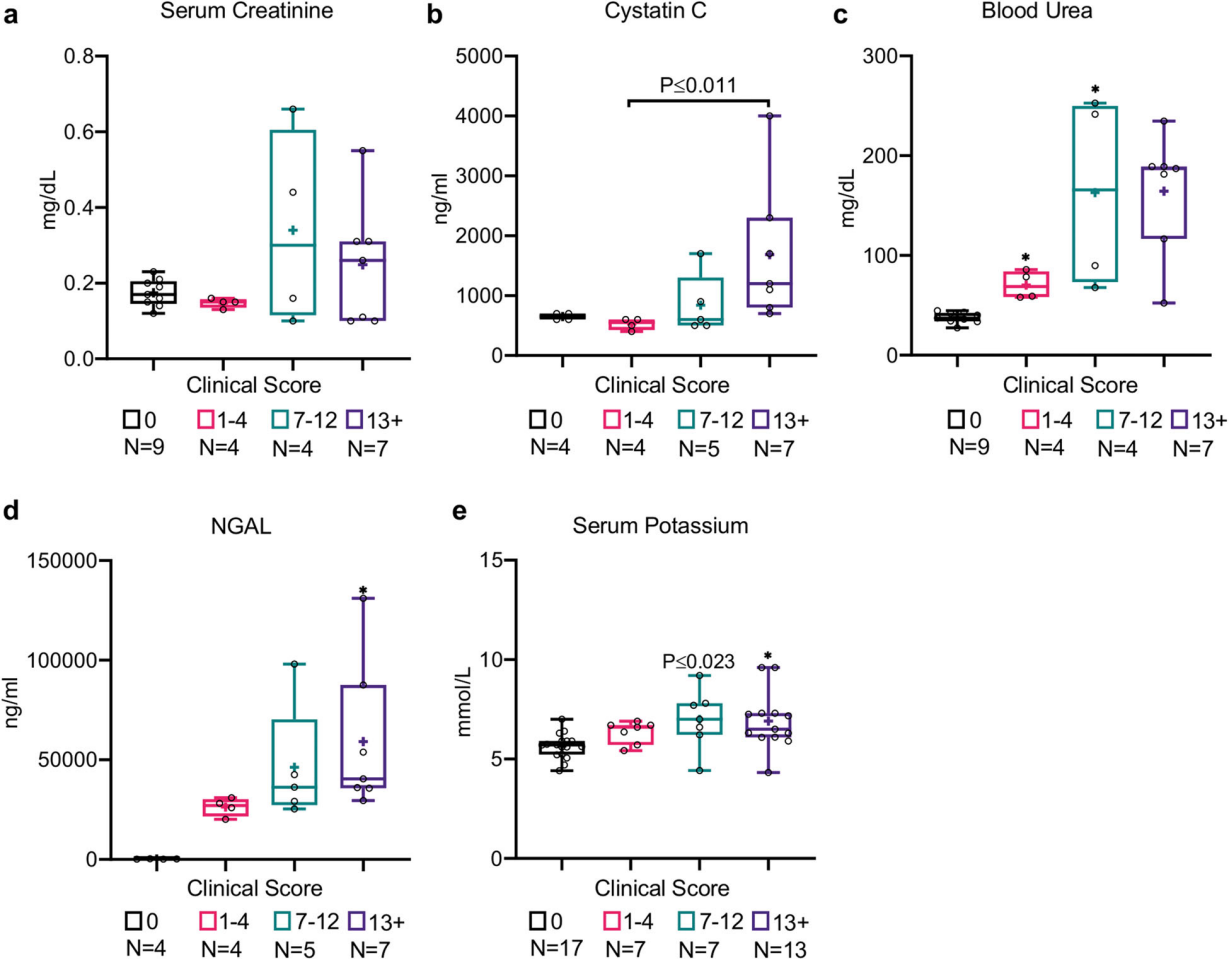
CLP mice display signs of renal dysfunction that correlates with sepsis severity. Renal dysfunction is indicated by increasing concentrations of **a** serum creatinine, **b** cystatin C, **c** blood urea, **d** NGAL, and **e** serum potassium. Data are presented as the median within the inter-quartile range (IQR); mean values are marked with a ‘+’ sign; error bars represent the minimum and maximum; group sizes (*N*) are indicated below their respective legends; *P ≤ 0.01, **P ≤ 0.001, ***P ≤ 0.0001 by the Kruskal–Wallis nonparametric ANOVA, with multiple-comparisons adjusted using Dunn’s test. *P* values above the bars indicate significant differences from the control group, and those above the brackets indicate the significant differences between the other two groups.

An exaggerated inflammatory response combined with cardiovascular dysfunction in sepsis can seriously damage renal function^34,35^. Therefore, renal dysfunction was evaluated by measuring creatinine and urea as well as newer markers, i.e., cystatin C and neutrophil gelatinase-associated lipocalin (NGAL). Although serum creatinine and cystatin C were elevated in CLP mice in comparison to naive mice, significant increases were seen only in moderately-to-severely septic mice (MSS of 7–12 and 13+, respectively), and not in mice with mild sepsis (MSS ≤ 4), which represent the early stage of sepsis (Fig. 2a, b). This observation probably indicates a relatively late effect. However, urea levels were significantly elevated in CLP mice with low and moderate clinical scores (Fig. 2c; *P* ≤ 0.01 for both groups), and strongly correlated with MSS (Table 1; ρ-Spearman = 0.8852). In contrast to the late effect on serum creatinine, NGAL was suggested to correlate well with AKI in sepsis model mice^36^. Indeed, NGAL serum concentration was dramatically increased, especially in CLP mice with severe sepsis (Fig. 2d; *P* ≤ 0.01; MSS of 13+), and strongly correlated with the clinical score (Table 1; ρ-Spearman = 0.7572). Together with a moderate but significant increase in serum potassium in CLP mice (Fig. 2e; *P* ≤ 0.01; MSS of 13+), these results are indicative of AKI.

### Markers for acute liver injury strongly correlate with the MSS in CLP mice

**Fig. 3:**
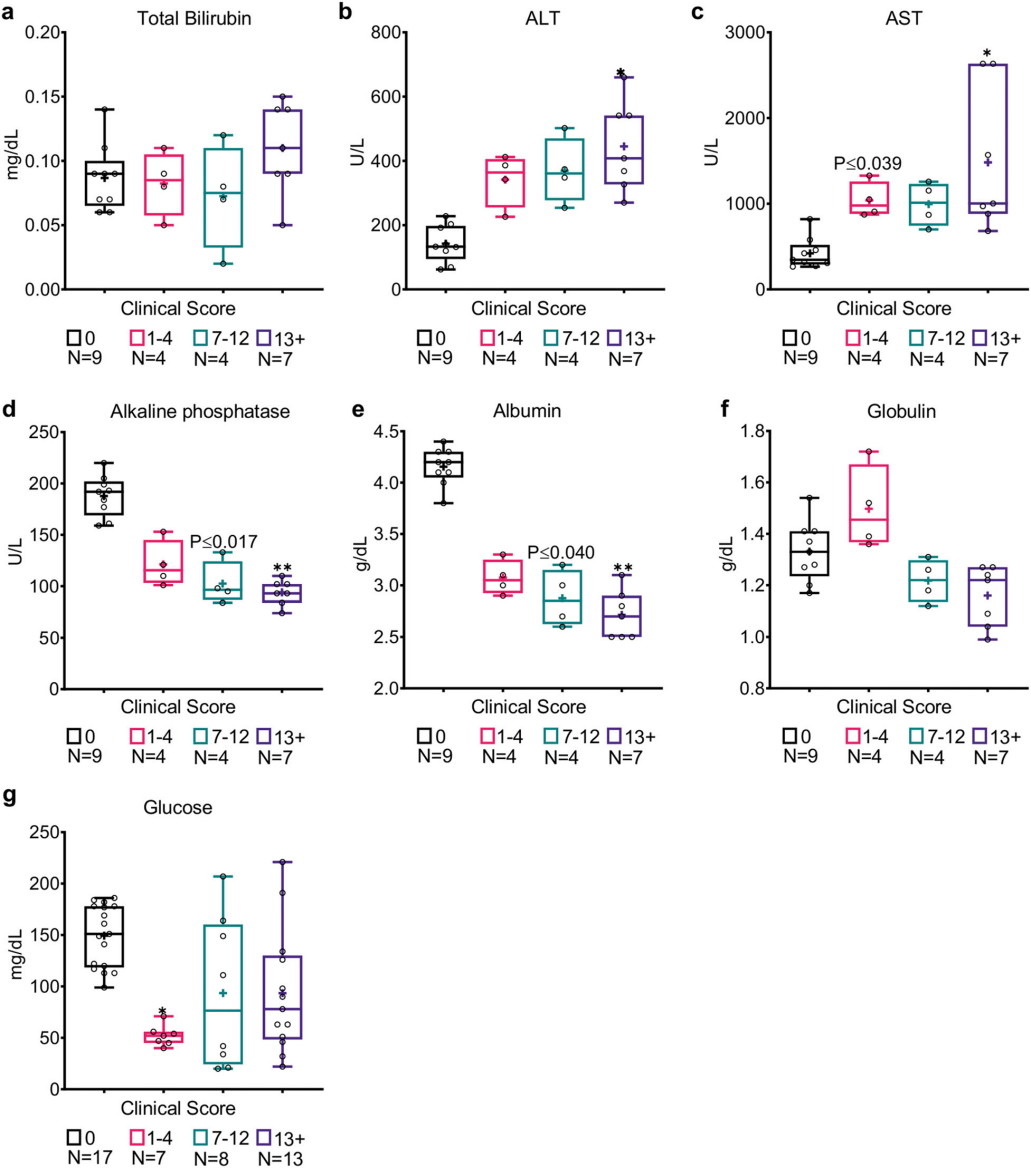
Markers for hepatic dysfunction strongly correlate with MSS in CLP mice. **a** Twenty-four hours post-CLP, mice with severe sepsis (MSS > 13) had a slight and insignificant (*P* > 0.93) increase in total bilirubin serum concentration, while **b** increases in ALT and **c** AST levels were significantly correlated with sepsis severity. **d** Alkaline phosphatase and **e** albumin levels were significantly decreased with sepsis severity, while **f** globulin serum concentrations were not significantly altered. **g** Glucose levels of septic mice, predominantly in mildly septic mice (MSS 1–4), were lower than those in naive mice. Data are presented as the median within the inter-quartile range (IQR); mean values are marked with a ‘+’ sign; error bars represent the minimum and maximum; group sizes (*N*) are indicated below their respective legends; *P ≤ 0.01, **P ≤ 0.001, ***P ≤ 0.0001 by the Kruskal–Wallis nonparametric ANOVA, with multiple-comparisons adjusted using Dunn’s test. *P* values above the bars indicate the significant differences from the control group, and those above the brackets indicate the significant differences between the two other groups.

Liver dysfunction occurs in almost 40% of sepsis patients^37^ and can be diagnosed by an increase of serum bilirubin and liver transaminases, and a decrease in albumin production^17,38–40^. CLP mice were shown to follow the same trend^26,29,33^. In our study, CLP mice with severe sepsis had a late non-significant increase of serum bilirubin (Fig. 3a; *P* > 0.93). Nevertheless, both aspartate transaminase (AST) and alanine transaminase (ALT) levels were significantly elevated in CLP mice compared with naive mice (Table 1; *P* ≤ 0.001), especially in mice with severe sepsis (Fig. 3b, c; *P* ≤ 0.01). The dramatic increases in AST and ALT were clearly reflected in the MSS (Table 1; ρ-Spearman = 0.7268 and 0.8216, respectively). A substantial release of liver transaminases that is not accompanied by a significant increase of bilirubin is typical of hypoxic hepatitis^38,40^, and may suggest this mechanism for acute liver injury.

The reduction of alkaline phosphatase (ALP) was most prominent in mice with moderate and severe sepsis (Fig. 3d; *P* ≤ 0.017 and *P* ≤ 0.001 for MSS of 7–12 and 13+, respectively). Twenty-four hours post-CLP, both total protein serum levels, and serum albumin levels had significantly dropped (Table 1; *P* < 0.0001). These decreased protein levels are probably attributed to liver dysfunction, since albumin (which is produced primarily in the liver) was decreased, but not globulin (Fig. 3e, f). Interestingly, glucose levels were significantly decreased, mainly in mildly septic mice (Fig. 3g; *P* ≤ 0.01 for MSS of 1–4), but also in general (Table 1; *P* ≤ 0.0001). This phenomenon may be partly related to gluconeogenesis dysfunction in the liver.

### Marked thrombocytopenia and lymphopenia in septic mice

**Fig. 4:**
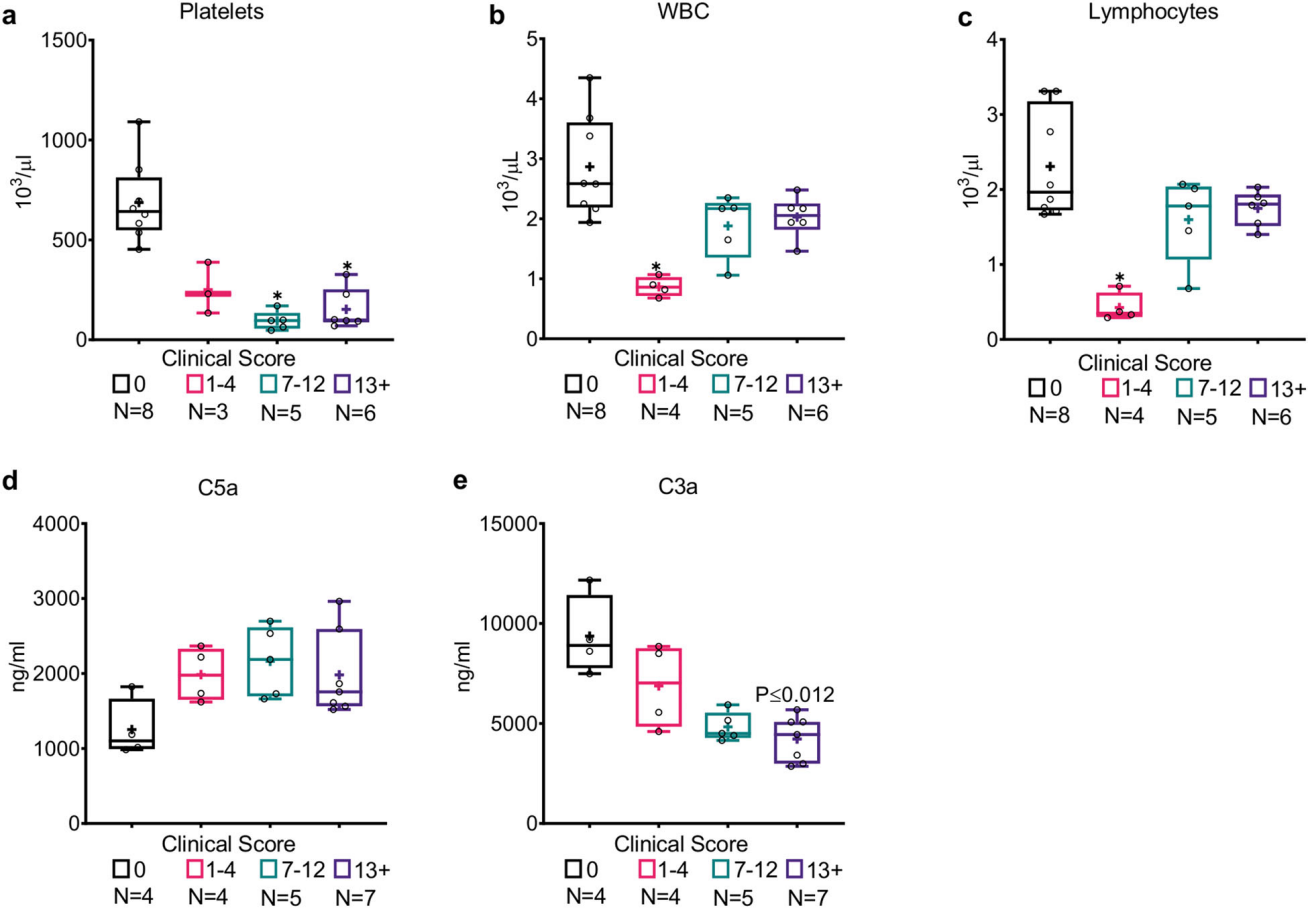
Marked thrombocytopenia and lymphopenia and aberrant complement activation in septic mice. **a** Twenty-four hours post-CLP, septic mice had significantly lower platelet count than naive mice. Decreased **b** WBC and **c** Lymphocyte counts in CLP mice, predominantly in mice with mild sepsis (MSS 1–4). **d** C5a serum concentration is higher in septic mice, regardless of their clinical score. **e** C3a serum concentration is lower in septic mice with correlation to clinical score. Data are presented as the median within the inter-quartile range (IQR); mean values are marked with a ‘+’ sign; error bars represent the minimum and maximum; group sizes (*N*) are indicated below their respective legends; *P ≤ 0.01, **P ≤ 0.001, ***P ≤ 0.0001 by the Kruskal–Wallis nonparametric ANOVA, with multiple-comparisons adjusted by using the Dunn’s test. *P* values above the bars indicate significant differences from the control group, and those above the brackets indicate significant differences between the other two groups.

The hematological system is the first and one of the most affected systems in sepsis. Hematological aberrations in sepsis patients include thrombocytopenia, lymphopenia, and neutropenia or neutrophilia, all of which are associated with poor outcomes^41–45^. CLP mice are no different^28,33,46,47^. Hematological dysfunction in septic mice was thus evaluated by total blood count, including red blood cells (RBC), platelets, white blood cells (WBC, both general and subpopulations), and other parameters (hemoglobin, hematocrit, and cell volume). As seen in Table 1, the most dramatic effect on the hematological system was a sharp decrease (−6.49× fold change) of CLP mice platelet count in comparison to naive mice (*P* < 0.0001). This thrombocytopenia was in strong correlation to MSS (ρ-Spearman = −0.7099), and was more prominent in mice with moderate and severe sepsis, whose median platelet counts were below 100 × 10^3^/µl (Fig. 4a; 95% CI range of 37–260 × 10^3^/µl). Further details of hematological and complement systems are presented in Table 1 and Fig. 4, and provided in the supplementary section.

### Allocetra-OTS effects on sepsis severity are associated with rebalancing metabolic changes

**Fig. 5:**
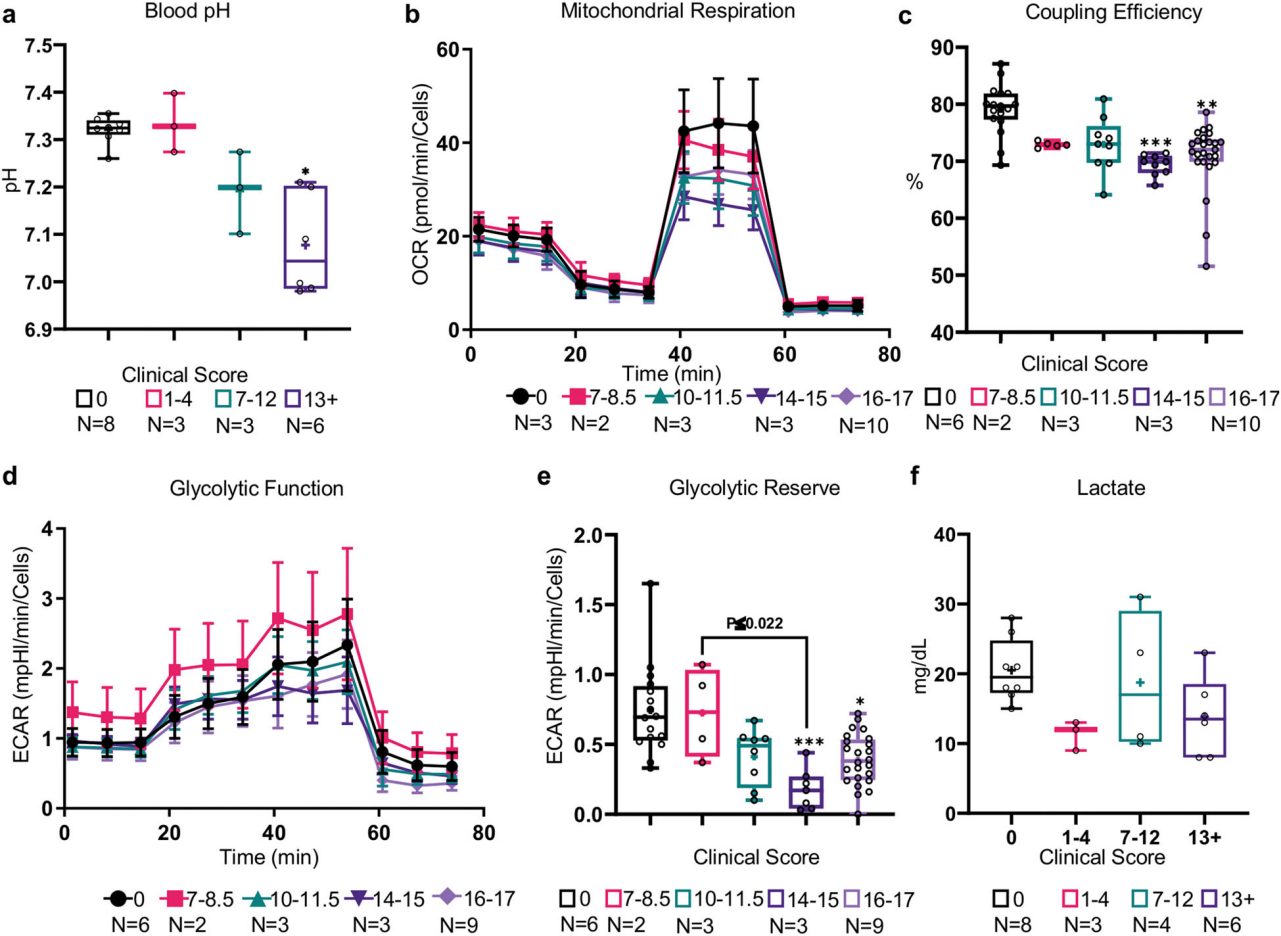
CLP mice present adverse metabolic changes. **a** Twenty-four hours post-CLP, blood pH was significantly decreased with sepsis severity. **b** OCR measurements of splenocytes from naive and CLP mice showed aberrant mitochondrial respiration, predominantly in severely septic mice (MSS > 10), which was manifested primarily by **c** a decreased coupling efficiency. **d** ECAR measurements of splenocytes from naive and CLP mice showed only mild changes in the general glycolytic function, which was slightly increased in moderately septic mice (MSS 7–8.5). **e** The glycolytic reserve of splenocytes in this assay was significantly decreased in severely septic mice (MSS > 14). **f** Blood lactate concentration was slightly lower in CLP mice. Data in (**a**), (**c**), (**e**), and (**f**) are presented as the median within the inter-quartile range (IQR); mean values are marked with a ‘+’ sign; error bars represent the minimum and maximum; data in (**b**) and (**d**) are presented as the mean ± standard deviation. Group sizes (*N*) are indicated below their respective legends; *P ≤ 0.01, **P ≤ 0.001, ***P ≤ 0.0001 by the Kruskal–Wallis nonparametric ANOVA, with multiple-comparisons adjusted using Dunn’s test. *P* values above the bars indicate the significant differences from the control group, and those above the brackets indicate the significant differences between the two other groups.

Because the pathogenesis of sepsis involves dramatic metabolic changes^48^, we were interested in exploring some of the major metabolic and bioenergetic markers of sepsis and examining their correlation to disease severity. CLP mice had a significantly lower blood pH than naive mice with a strong inverse correlation to clinical score (Table 1; *P* ≤ 0.0089, ρ-Spearman = −0.7792) the median blood pH of mice with severe sepsis compared with naive mice was even lower (Fig. 5a; 7.044 and 7.325, respectively). As noted in Fig. 3g, glucose levels were significantly decreased in septic mice, as previously described in late and severe stages of sepsis in mouse models of endotoxemia or CLP^29^.

In order to further explore the metabolic changes, we performed bioenergetics analysis to measure the oxygen consumption rate (OCR) and extracellular acidification rate (ECAR) of freshly isolated splenocytes from naive- and CLP mice. The results are shown in detail in Fig. 5, Supplementary Table 2, and in the Supplementary Material. In agreement with the lack of increasing glycolysis in severely septic mice, lactate was not elevated (Fig. 5f), as may be expected, perhaps reflecting the apparent inability of splenocytes from septic mice to shift from attenuated mitochondrial respiration to the glycolysis pathway in severe sepsis, and their failure to meet the energy demands of the immune system. Apoptotic cell treatment dramatically shifted MSS to levels where both the nonfunctioning mitochondria and glycolysis moved toward normal functional levels, and this effect may be an additional mechanism by which apoptotic cells shift the immune response toward homeostasis.

### Adding Allocetra-OTS to the conventional fluid resuscitation and ertapenem antibiotic treatment significantly increased the survival of CLP mice

**Fig. 6:**
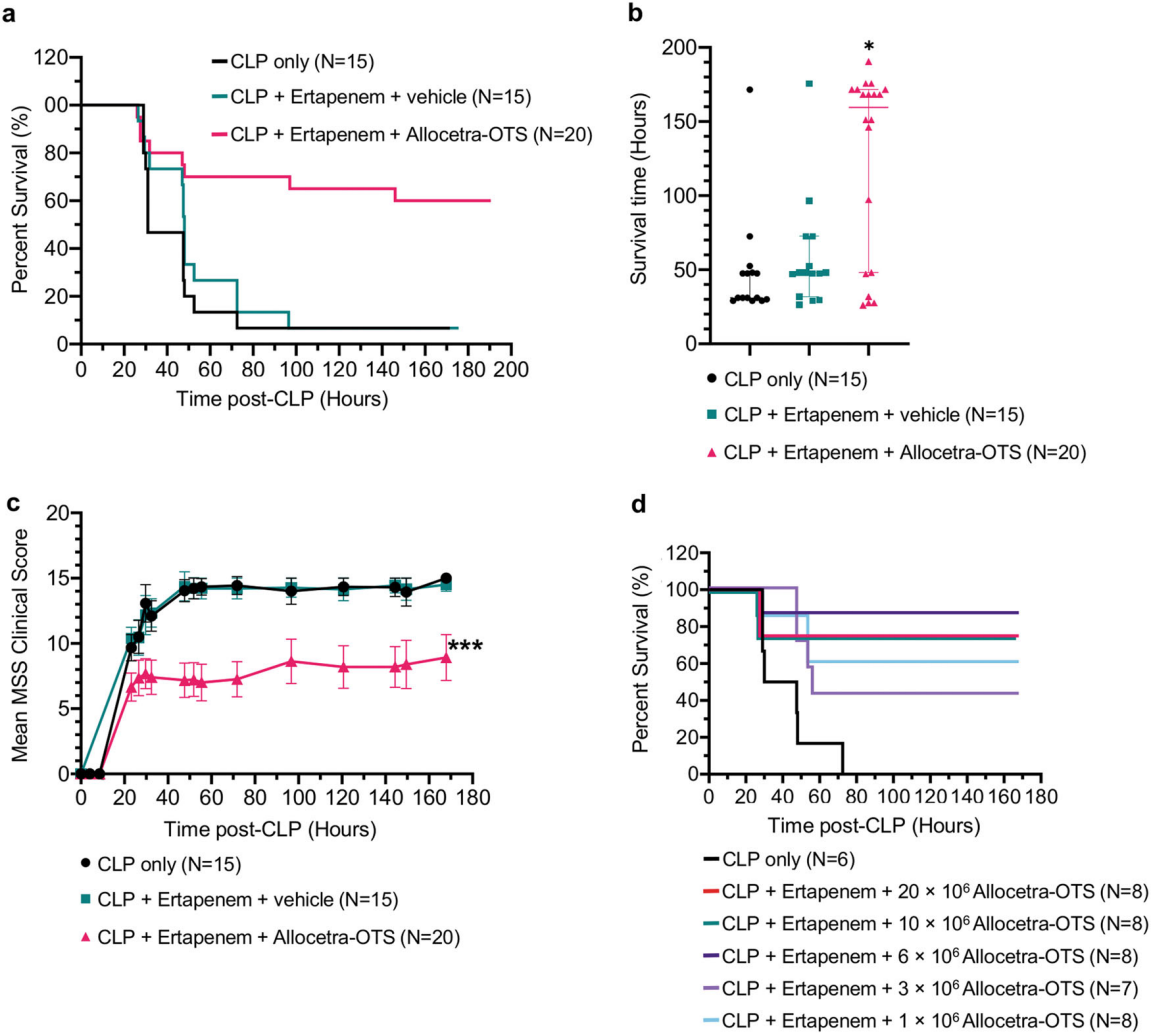
Beneficial effects of Allocetra-OTS on CLP mice. Four hours post CLP mice were i.v. injected with ertapenem and either Hartmann’s solution (vehicle) or 20 × 10^6^ Allocetra-OTS (unless otherwise indicated). Mice were monitored for well-being and euthanized when MSS > 15. **a** Kaplan–Meier survival curves of CLP mice either treated with ertapenem + vehicle or ertapenem + Allocetra-OTS. **b** Median survival time was increased in Allocetra-OTS-treated mice. Error bars represent the 95% CI; *P ≤ 0.01 by the Kruskal–Wallis nonparametric ANOVA, with multiple-comparisons adjusted using Dunn’s test. **c** The mean MSS was decreased in Allocetra-OTS-treated mice. Error bars represent the standard error; ***P ≤ 0.0001 by ordinary one-way ANOVA of the non-linear curve fits. **d** Kaplan–Meier survival curves of CLP mice treated with ertapenem + varying dosages of Allocetra-OTS. The numbers of mice in each group (*N*) are indicated beside their respective legends.

Since apoptotic cells were shown to bring an exaggerated cytokine/chemokine response back to homeostasis^20^, we envisaged treating CLP-induced septic mice with Allocetra-OTS to try rebalancing the immune response as a potential therapy for sepsis. 15/16 mice (94%) in the control group (CLP mice with vehicle injection only) died of sepsis 24–72 h after CLP. Compared with the CLP control group, ertapenem treatment with vehicle control (*n* = 15) had no significant effect on mouse survival, with only a slightly higher median survival (*P* > 0.99; 31 and 48 h, respectively), and similar mortality of 93%. Allocetra-OTS treatment combined with ertapenem significantly prolonged the survival of the mice following CLP-induced sepsis (Fig. 6a; *P* ≤ 0.0005, log-rank test). Among the mice treated with Allocetra-OTS and ertapenem, 8/20 (40%) died within 29–146 h after CLP; however, the majority of the mice remained alive at the end of the experiments 6–8 days post-CLP, with significantly increased median survival time of 160 h (Fig. 6b; *P* ≤ 0.0074, Kruskal–Wallis nonparametric ANOVA, multiple-comparisons adjusted with Dunn’s test; 95% CI: 48–172 h).

### Allocetra-OTS attenuates sepsis severity

Murine survival had a strong reverse-correlation with clinical score (r-Pearson −0.924; *P* < 0.0001). Treatment with Allocetra-OTS and ertapenem substantially attenuated the appearance of clinical symptoms. The final MSS of Allocetra-OTS-treated mice was significantly lower than those of the CLP control group and those of mice treated with ertapenem alone (Fig. 6c; MSS plateau values of 7.78, 14.81, and 14.37, respectively; *P* < 0.0001, ordinary one-way ANOVA).

A dose-dependent beneficial effect of Allocetra-OTS was also observed. CLP mice treated with Allocetra-OTS doses between 1 × 10^6^ and 20 × 10^6^ cells per mouse survived 42.85–87.5% longer than vehicle-treated CLP mice (Fig. 6d; *P* ≤ 0.0115, log-rank test). Although even low doses of 1 and 3 million Allocetra-OTS cells per mouse had a clear effect in severe sepsis, robust effects were seen only when using 6 million Allocetra-OTS cells or more. Doses of 3–6 million Allocetra-OTS cells were not examined.

### Allocetra-OTS effects on sepsis severity are achieved by rebalancing the immune response

**Fig. 7:**
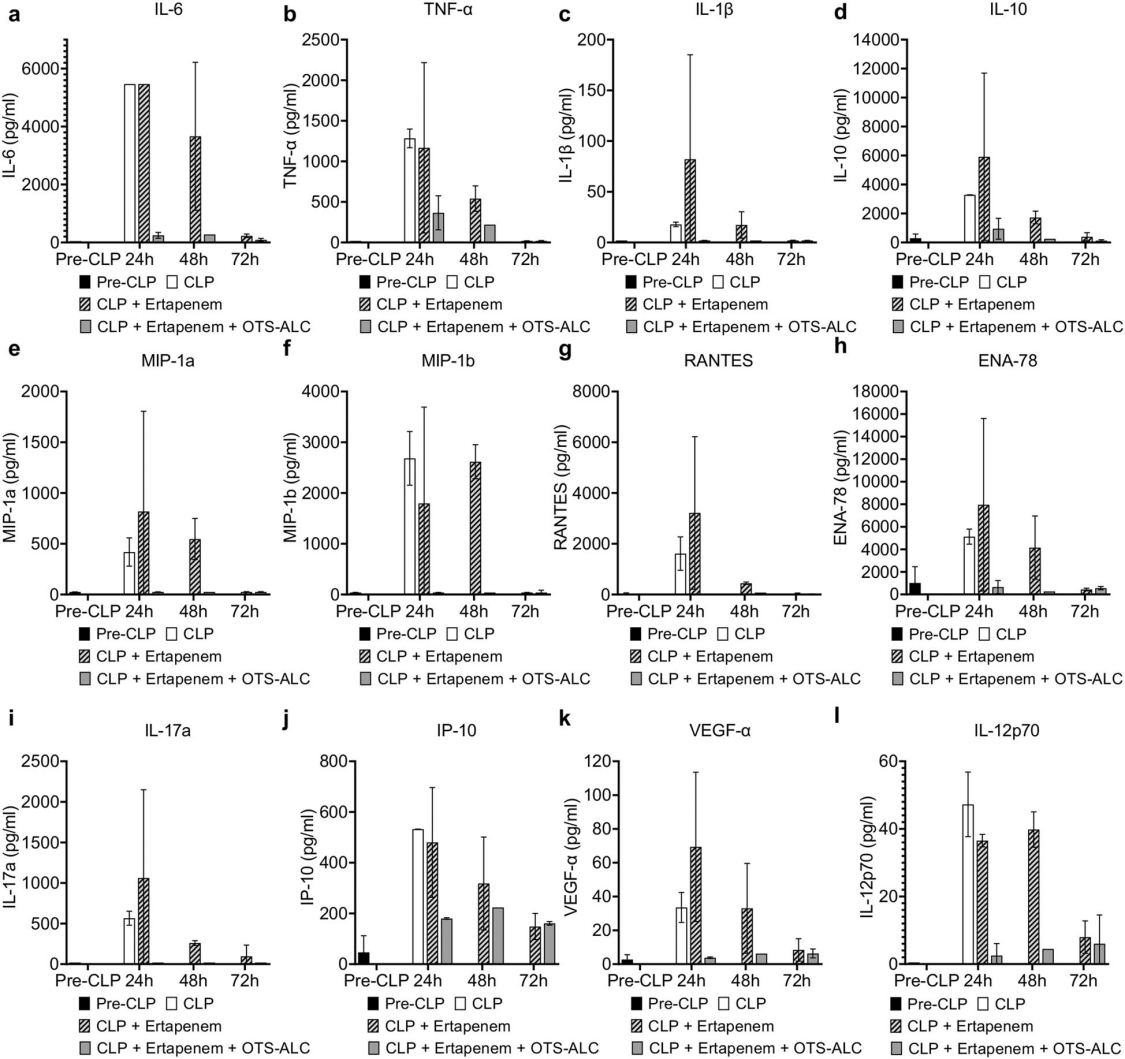
Allocetra-OTS treatment attenuates cytokine/chemokine release following CLP. Blood samples were taken from C57BL/6 mice before CLP and 24, 48, and 72 h post-CLP, after treatment with either ertapenem or a combination of ertapenem + Allocetra-OTS (OTS-ALC). Untreated CLP mice did not survive past 24 h; therefore, data are not shown. **a**–**l** Cytokine/chemokine levels measured by Luminex. Data are presented as the mean ± standard deviation.

As summarized in Supplementary Table 3, 33 different cytokine and chemokine levels were elevated 24 h post-CLP in CLP mice compared with naive C57BL/6 mice. Interestingly, while treatment with ertapenem antibiotics alone had no beneficial effects on cytokine or chemokine levels, combined treatment with ertapenem and Allocetra-OTS attenuated and even abolished cytokine and chemokine release at 24 h and even 48 h after sepsis induction (Fig. 7). The cytokine storm rebalancing effect of Allocetra-OTS corresponded well with the beneficial effects of Allocetra-OTS plus ertapenem on murine survival and sepsis severity. These findings strongly suggest that Allocetra-OTS confers its effects by breaking the exaggerated cytokine storm and rebalancing the immune response.

## Discussion

Sepsis is generally initiated by simultaneous recognition of either PAMPs or DAMPs by various receptors, inducing a complex intracellular signaling system like the inflammasome^49,50^, with redundant and complementary activities. The complementary nature of the pathways explains the overlapping yet unique early inflammatory response to a variety of infections, that include inflammatory syndrome, immune suppression, and possibly tissue injury^11,12,16^.

In this study, we employed a CLP-induced murine model for sepsis treated with the combination of antibiotics and fluid resuscitation to successfully emulate human sepsis. This model simulated severe sepsis with acute multiple organ dysfunction. The MSS method, adopted from Shrum et al.^30^, was used to assess disease severity in tandem with multiple organ analyses, and the correlation between parameters of organ dysfunction and disease severity was assessed. Indeed, CLP mice had low blood pressure, poor cardiac output, and lung dysfunction, as well as AKI, ALI, and thrombocytopenia that correlated with their clinical score. These multi-organ failures are well documented in patients with severe sepsis and septic shock^51–53^, and in murine sepsis models^28,33,54,55^, and are the basis for the sequential organ failure assessment (SOFA) score calculation^56^. In sepsis and septic shock patients, upregulation of many cytokines, including IL-1β, IL-6, IL-8, IL-10, MCP-1, and G-CSF, was associated with increased mortality and positively correlated with patients’ SOFA scores^14,57^. Likewise, in our study the CLP mice had elevated cytokine secretion that corresponded with their MSS.

Apoptotic cells were shown to have a beneficial effect on cytokine storms with downregulation of both anti- and pro-inflammatory cytokines derived from PAMPs and DAMPs, both in animal models and in in vitro and in vivo human studies^20,21^. Clearance of apoptotic cells allows immune homeostasis. As shown here, this generally leads to a non-inflammatory state for both macrophages and DCs and contributes to the maintenance of peripheral homeostasis of almost any immune-triggered mechanism in sepsis.

The pathogenesis of sepsis is strongly related to vast changes in metabolic homeostasis, which was indeed compromised in CLP-related sepsis and significantly recovered during apoptotic cell treatment. Recent studies have provided evidence for metabolic switching from oxidative phosphorylation to aerobic glycolysis to meet the increasing energy demands of activated leukocytes in inflammation and sepsis^58,59^.

During the acute phase of sepsis, the shift from oxidative phosphorylation to aerobic glycolysis (the Warburg effect) is an important mechanism of host defense^48^. Immunometabolic paralysis was suggested as a therapeutic target for the treatment of sepsis^48,59^. Indeed, defects in energy production by oxidative phosphorylation were very severe and could not be rescued by the compromised alternative glycolysis pathway. Weis et al.^60^ have shown that liver glucose production in response to bacterial infection is essential to establish disease tolerance. The authors suggest that impaired liver gluconeogenesis is related to lethal hypoglycemia in response to acute infection.

Interestingly, during efferocytosis, multiple transcriptional programs are modified, resulting in decreased pro-inflammatory gene expression with increased actin rearrangement, increased expression of cell motility genes, and anti-inflammatory mediators, but also increased expression of anti-apoptotic and cell growth genes as well as intracellular signaling mechanisms^61^ that may avoid phagocyte programmed cell death. During apoptotic cell internalization, simultaneously, ERK phosphorylates paxillin, and phosphorylated paxillin serves as a scaffold for FAK to activate PI3K for RAC activation^61^. Furthermore, apoptotic cell uptake increases glycolysis within phagocytes that contribute to actin polymerization and the continued uptake of corpses, and increases lactate released via SLC16A1, promoting the establishment of an anti-inflammatory tissue environment^61^. Collectively, these effects suggest that apoptotic cells affect phagocytes via increased glycolysis and a pro-homeostatic immune response, leading to organ preservation and a beneficial effect during sepsis.

In summary, using the CLP model, we showed that a single dose of Allocetra-OTS not only significantly increased murine survival but did so in a dose-dependent manner. Furthermore, the combined treatment with ertapenem antibiotic and Allocetra-OTS significantly attenuated disease severity by almost 50%, leading to a 10-fold increase in survival. It should be emphasized that treatment aiming to modulate the immune response is not administered instead of antibiotic treatment, fluid resuscitation, and vasopressors. Rather apoptotic cells are an adjuvant and complementary treatment that rebalances the immune response. The properties of apoptotic cells that lead to their successful use as a therapeutic modality in sepsis may also be relevant in various autoimmune and autoinflammatory diseases, cytokine storms, organ transplantation, and graft-versus-host disease (GVHD).

## Methods

### CLP procedure

C57BL/6 female mice were operated under general isoflurane (2%) anesthesia. Analgesics were administered by subcutaneous injection (SC). Perforation of the cecum was followed by the release of fecal material into the peritoneal cavity. Mice that died during the first 24 h after surgery were considered as perioperative mortality and were immediately excluded from the experiment, as their death was due to perioperative complications and not to sepsis. (Further details are provided in supplementary methods.)

### Allocetra-OTS

An enriched mononuclear cell fraction was collected via leukapheresis from healthy, eligible donors who had signed informed consent forms approved by the Ethical Committee (Hadassah-Hebrew University # HMO-0066-18). For the preparation of Allocetra-OTS, cryopreserved cells were thawed, washed, and resuspended with apoptosis induction media containing methylprednisolone. Apoptosis and viability of Allocetra-OTS were determined using Annexin V and PI staining (Medical & Biological Laboratories, Nagano, Japan) by flow cytometry (FACSCalibur, Becton Dickinson, Franklin Lakes, NJ, USA, supplementary Fig. 1). In general, 20 × 10^6^ Allocetra-OTS cells were injected IV per mouse.

### Antibiotic treatment

Mice received 75 mg/kg ertapenem IP immediately after Allocetra-OTS or vehicle administration and then every 24 h for 3 days.

#### Blood pressure

Mice were measured for blood pressure using a CODA noninvasive blood pressure system (Kent Scientific, Torrington, CN, USA).

#### Lungs and organ dysfunction tests

Twenty-two to twenty-four hours after CLP, mice were weighed, assigned an MSS, and sacrificed. Lungs were harvested and weighed, and lung-to-body weight ratios were calculated. Organ dysfunction was evaluated.

#### Hematology

Two hundred and fifty microliters of blood was collected into EDTA tubes (MiniCollect, Greiner Bio-One, Kresmünster, Austria), tubes were rotated to prevent blood clotting and kept at 4 °C. Hematology analysis was performed by AML laboratories (Herzliya, Israel).

#### Biochemistry

Biochemistry analysis of mouse serum was performed by AML laboratories (Herzliya, Israel).

#### Analysis of cytokines/chemokines, NGAL and Cystatin C, and complement components

Serum cytokine/chemokine, NGAL and Cystatin C measurements were performed using multiplex ELISA (Luminex), and serum complement components (C5a, C3a) were measured by sandwich ELISA, as detailed in the Supplementary Methods.

#### 2D Echocardiography

Twenty-four hours after CLP, naive mice (*n* = 5) or ertapenem-treated CLP mice (*n* = 10) were anesthetized with isoflurane and their left ventricle (LV) function was evaluated by echocardiography using a high-resolution imaging system (Vevo 770, Visual Sonics, Canada), as detailed in the Supplementary Methods.

#### Bioenergetics analysis

Twenty-four hours after CLP, mice splenocytes were isolated and their glycolytic and mitochondrial activities were assayed with the XF96 Extracellular Flux Analyzer (Seahorse Bioscience, North Billerica, MA, USA) using the Glyco and Mito Stress kits (Agilent, Santa Clara, CA, USA), as described in detail in the Supplementary Methods.

### Statistics

Differences between groups were examined for statistical significance using the Mann–Whitney nonparametric test. Differences between multiple groups were examined for statistical significance using Kruskal–Wallis one-way analysis of variance (nonparametric ANOVA) with multiple-comparisons adjusted by using the Dunn’s test. Lung/body weight ratio was examined using ANOVA. Correlation of any parameter to clinical score was evaluated by a Spearman’s rank correlation coefficient, with a coefficient higher than 0.7 or lower than −0.7 being a strong correlation. All statistical analyses were done using GraphPad Prism (San Diego, CA, USA). Survival analysis was performed according to the Kaplan–Meier method. A Log-rank statistical test was performed using GraphPad Prism.

### Study approval

The experimental procedures for the animal studies were approved by the Institutional Animal Care and Use Committee of the Hebrew University Medical School.

## Supplementary information

Supplementary Materials

Supplementary Figure Legends

Supplementary Figure 1
