# Supplementary material for: Apoptotic cell therapy for cytokine storm associated with acute severe sepsis
Source: Cell Death Dis. 2020 Jul 15;11(7):535. doi: 10.1038/s41419-020-02748-8 (PMC7363887; doi:10.1038/s41419-020-02748-8)
Supplement: Supplementary file 1 — Supplementary Materials [file 41419_2020_2748_MOESM1_ESM.docx]

**Supplementary Materials**

**Apoptotic cell therapy for cytokine storm associated with Acute Severe Sepsis**

Netanel Karbian^1^, Avraham Abutbul^2^, Raja el-Amore^1^, Ran Eliaz^3^, Ronen Beeri^3^, Barak Reicher^4^ and Dror Mevorach^1,5*^

**Content:**

**Supplementary Results**

**Supplementary Methods**

**Supplementary References**

**Supplementary Tables 1-3**

**Supplementary Fig. 1**

**Supplementary Results**

***Evaluation of the MSS clinical scoring system as a surrogate indicator for organ dysfunction in CLP mice***. Sepsis elicits dysregulated immune responses, which in turn dramatically disrupt the physiological homeostasis of vital organs including the kidney, liver, lungs, and heart. This imbalance often rapidly escalates into MODS, which is usually associated with poor outcomes^1,2^.

CLP mice were compared to naïve mice (MSS of 0; N=21). The CLP mice were divided into three sub-groups, based on their clinical scores: 1–4 (mild sepsis), 7–12 (moderate sepsis), and 13+ (severe sepsis). 24 hours post-CLP, most mice exhibited severe clinical signs, with a median MSS of 13 (95% CI of 9–14), indicating moderate to severe sepsis (Fig. 1a).

To study the cardiac function of CLP mice, 24 hours post-CLP the left ventricle (LV) of naïve- (n=5) and ertapenem-treated CLP mice (n=10) was imaged by echocardiography, and various structural and functional cardiac parameters were tested for their correlation with the clinical score (Supplementary Table 1).

***Markers for acute liver injury strongly correlate with the MSS clinical score in CLP mice.*** Alkaline phosphatase (ALP) may also be elevated in human sepsis patients, possibly as an anti-inflammatory and anti-microbial agent with a protective function against acute kidney injury^3,4^. Indeed, both in severe sepsis and in this model, and with severe AKI, ALP serum concentrations are substantially reduced in comparison to naïve mice, with a strong inverse correlation to the MSS (Table 1; p<0.0001, ρ Spearman=-0.8432). ALP consists of several endogenous metalloenzymes found in serum and in multiple organs throughout the body, including bone, liver, intestine, and kidney^5^. ALP is well established as a biomarker of liver and bone disease, but its physiological roles remain incompletely understood. Recent evidence points towards a potential protective effect of ALP in the mitigation of AKI through dephosphorylation of nephrotoxic molecules, including extracellular adenine nucleotides^6–10^ and endotoxins^4,9,10^. Less is known about ALP serum concentration in CLP mice, although a few studies demonstrated an increase of ALP following CLP in mice^11,12^.

**The hematological and complement system**. There were no differences between CLP mice and healthy mice in RBC, hemoglobin, hematocrit, or cell volume (Table 1; p=NS). A slight neutrophilia was also observed in septic mice, with a moderate inverse correlation to clinical score (Table 1; p≤0.0382; ρ Spearman=-0.4531). However, total WBC count in septic mice was significantly lower than that in healthy mice (Table 1; p≤0.0017). Interestingly, the lowest WBC count was in mice with mild sepsis rather than mice with severe sepsis (Fig. 4b; p≤0.01 for MSS of 1–4). This was mainly due to lower lymphocytes counts in septic mice (Table 1; p≤0.0275), which was mainly attributed to severe lymphopenia in mildly septic mice (Fig. 4c; p≤0.01 for MSS of 1–4).

***Aberrant complement activation pattern following CLP.*** The complement immune system is a major responder to infection, and as such is highly activated in sepsis^13^; however, excessive activation may lead to deleterious effects^14–16^. Accordingly, high levels of the complement proteins C3a and C5a were detected in sepsis patients^17–20^. While excessive generation of C5a causes harmful effects such as impaired neutrophil function and hyper-inflammation^21^, some cohort studies of sepsis patients showed links between higher C3a levels and survival^22^ or C3 depletion and high mortality^23,24^. The opposing protective effects of C3 and the harmful effects of C5 have been well-demonstrated in murine sepsis models of C3^-/-^ and C5^-/-^ or C3aR^-/-^ and C5aR^-/-^ after CLP. In these studies, C3-deficient mice had the poorest survival in comparison to WT and C5-deficient animals^15^. Furthermore, C5aR^-/-^ mice were resistant to Gram-negative bacteremia while C3aR^-/-^ were much more sensitive to this infection^25^. The effect of sepsis on the complement immune system was evaluated by measuring the serum concentration of C3a and C5a, 24 hours post-CLP. As expected, C5a serum concentrations were elevated in CLP mice (Table 1; p≤0.0219); however, as an early event, it did not correlate with MSS (Table 1). As seen in Fig. 4d, C5 elevation was an early event and therefore was elevated in all CLP mice, regardless of their clinical score. Interestingly, C3a levels were significantly decreased in CLP mice, and strongly correlated with MSS clinical score (Table 1; p≤0.0029, ρ Spearman= -0.7183). This decrease was the most significant in mice with a severe clinical score (Fig. 4e; p≤0.012 for MSS of 13+).

***Allocetra-OTS effects on sepsis severity are associated with rebalancing metabolic changes.***

In order to further explore the metabolic changes, we performed bioenergetics analysis to measure the oxygen consumption rate (OCR) and extracellular acidification rate (ECAR) of freshly isolated splenocytes from naïve- and CLP mice. These levels directly reflect mitochondrial function and glycolysis. The general mitochondrial respiration of splenocytes from CLP mice was compromised, especially in mice with severe clinical scores (Fig. 5b), as manifested by significantly decreased maximal respiration (Supplementary Table 2; P≤0.022), a mildly increased proton leak, and reduced spare respiratory capacity (Supplementary Table 2; p=n.s.). Significantly reduced ATP production and coupling efficiency were also strongly and inversely correlated with the clinical score (Supplementary Table 2; p≤0.001, ρ Spearman <-0.7, Fig. 5c). ECAR analysis of the same cells revealed a different pattern of glycolytic function; interestingly, while moderately septic mice (MSS clinical scores of 7–8.5) increased their glycolytic activity, severely septic mice (MSS >10) had reduced glycolysis (Fig. 5d). The only glycolytic parameter that was in correlation with the clinical score was the glycolytic reserve (Supplementary Table 2, Fig. 5e; p=n.s., ρ Spearman= -0.499).

***Adding Allocetra-OTS to the conventional fluid resuscitation and ertapenem antibiotic treatment significantly increased the survival of CLP mice*.** Mice underwent CLP procedures to induce sepsis, as detailed in Methods. Perioperative survival of mice from the CLP procedure using the isoflurane anesthesia machine was considered high; only 3 out of 54 mice (5.5%) died during the first 24 h after the procedure (interval of 6.5–20h) and they were excluded from the study.

15 of 16 mice (94%) in the control group (CLP mice with vehicle injection only) died of sepsis 24–72 hours after CLP. Compared to the CLP control group, ertapenem treatment with vehicle control (n=15) had no significant effect on mouse survival, with only a slightly higher median survival (P>0.99; 31h and 48h, respectively), and similar mortality of 93%. Allocetra-OTS treatment combined with ertapenem significantly prolonged the survival of the mice following CLP-induced sepsis (Fig. 6a; p≤0.0005, log-rank test). Among the mice treated with Allocetra-OTS and ertapenem, eight of 20 (40%) died within 29–146 hours after CLP; however, the majority of the mice remained alive at the end of the experiments 6–8 days post-CLP, with significantly increased median survival time of 160h (Fig. 6b; p≤0.0074, Kruskal-Wallis nonparametric ANOVA, multiple-comparisons adjusted with Dunn’s test; 95% CI: 48h–172h).

**Summary of results.**

Previously, it was suggested that the dramatic effect of a single apoptotic cell infusion on sepsis progression was attributed rebalancing of the immune systems via interaction with monocytes, macrophages, and DCs^26,27^. Taken together, these results show that in this CLP model of severe sepsis, the majority of significantly altered parameters of organ dysfunction strongly correlated with the MSS clinical score. These markers cover five of the main systems and organs that are damaged in sepsis (brain was not examined). Furthermore, based on the large area under the curves (AUC) of their ROCs, all markers that had a strong correlation with the clinical score can be used for prognosis in severe sepsis (Table 1; AUC>0.840). Therefore, the MSS clinical scoring system strongly reflects the pathophysiological status of the mice, and as such was used to evaluate the efficacy of our Allocetra-OTS treatment. The results show a significant improvement in organ function and survival in response to apoptotic cells that are suggested to be related to driving phagocytic cells towards homeostasis, rebalancing cytokine and chemokine secretion, and improvement of their metabolic dysfunction.

**Supplementary Methods**

***Mice.*** C57BL/6 female mice, 10–13wk old, were purchased from ENVIGO (Jerusalem, Israel). Mice were kept in an SPF animal facility in compliance with institutional IACUC guidelines. They were weighed daily and monitored 2–3 times a day for clinical signs and determination of the MSS clinical score^28^. The endpoint was defined as a total score of 15 or a maximum score in one of the categories in the table.

***CLP procedure.*** The procedure was performed as follows*:* the mice were operated under general isoflurane (2%) anesthesia. Analgesics were administered by subcutaneous (SC) injection of tramadol 5 mg/kg in 0.1ml of prewarmed 0.9% saline solution. After opening the abdomen of the mouse via a midline incision, the cecum was exposed and ligated 75% above its distal end with a 4-0 silk suture. Following ligation, the cecum was perforated twice with a 19-gauge needle, using the through-and-through technique. Perforation of the cecum was followed by the release of fecal material into the peritoneal cavity. Afterward, the cecum was returned to the peritoneal cavity and 0.5ml of prewarmed saline was administered to the peritoneal cavity, which was subsequently sutured with a 4-0 vicryl suture. The skin was then closed with 9mm clips and mice were placed under a heating lamp to recuperate. They received tramadol every 12h for the first 36h after the procedure. Mice that died during the first 24h after surgery were considered as perioperative mortality and were immediately excluded from the experiment, as their death was due to perioperative complications and not to sepsis. Mice were bled through the retro-orbital sinus (venous blood). Naïve mice were bled under isoflurane analgesia; CLP mice were bled without analgesia due to the concern of death.

***Allocetra-OTS.*** An enriched mononuclear cell fraction was collected via leukapheresis from healthy, eligible donors following signing an informed consent approved by the Ethical Committee (Hadassah-Hebrew University # HMO-0066-18). Following the completion of apheresis, cells were washed and resuspended with freezing media. Cells were then gradually frozen and transferred to liquid nitrogen for long-term storage. For the preparation of Allocetra-OTS, cryopreserved cells were thawed, washed, and resuspended with apoptosis induction media containing methylprednisolone. Apoptosis and viability of Allocetra-OTS were determined using Annexin V and PI staining (Medical & Biological Laboratories, Nagano, Japan) by flow cytometry (FACSCalibur, Becton Dickinson, Franklin Lakes, NJ, USA, supplementary Fig. 1). In general, 20×10^6^ Allocetra-OTS cells were injected IV per mouse. The CLP procedures required about 20 minutes per mouse and the overall procedure lasted for about 5 hours. Allocetra-OTS was injected into each mouse 4h after its procedure had ended. Control mice received a Hartmann vehicle solution at the same time point. For dose calibration of Allocetra-OTS cells, each mouse received 1, 3, 6, 10, or 20×10^6^ cells.

**Blood pressure.** Blood pressure was measured the day before the CLP procedure to establish a baseline, and again 4 hours following the CLP procedure. For each mouse, 3 measurements were made at each time point to provide more accurate data.

**Blood gas.** 100µl blood was collected using heparin-coated capillary tubes (Paul Marienfeld, KG, Lauda-Königshofen, Germany) and immediately tested using the STAT profile prime machine (Nova Biomedical, Waltham, MA, USA).

***Serum sample collection.*** At the indicated times ~500µl blood was collected in a pre-labeled Eppendorf tube and left for 30min to allow clotting. The samples were centrifuged at 1800g (3000rpm) for 10min at 4°C, 200µl. The serum was transferred to a new pre-labeled Eppendorf tube and kept at 4°C. Excess serum was stored at -80°C.

**Biochemistry.** Biochemistry analysis of mouse serum was performed by AML laboratories (Herzliya, Israel).

***Luminex® analysis of cytokines/chemokines* NGAL and Cystatin C.** Serum cytokine/chemokine measurement was performed using the Luminex MAGPIX system (Luminex, Austin, TX, USA), and analysis was performed with Milliplex software (MilliporeSigma/Merck, Darmstadt, Germany). NGAL and Cystatin C were tested by the Luminex Multiplex kit (MKI2MAG-P4k). All reagents were provided with the kit, and all reagents were prepared according to the manufacturers’ protocols. The assays were performed in 96-well plates according to the protocol provided. Plate reading was performed with the Luminex MAGPIX system and analyzed using Milliplex software. The raw data was measured as mean fluorescence intensity (MFI) and the concentration of each analyte for each sample was calculated using a 4- or 5-parameter logistic fit curve generated for each analyte from the seven standards. The lower limit of quantification (LLOQ) was determined using the lowest standard that was at least three times above background. The calculation of the LLOQ was performed by subtracting the MFI of the background (diluent) from the MFI of the lowest standard concentration and back calculating the concentration from the standard curve.

***ELISA analysis of complement components* (C5a, C3a).** Serum complement components were tested by sandwich ELISA kits: C3a (TECO, TEI038) and C5a (EA100633, OriGene, Rockville, MD, USA). All reagents were provided with the kits and prepared according to the manufacturers’ protocols. Assays were performed in 96-well plates according to the protocols provided. OD plate reading was performed with the Infinite F50 (TECAN, Männedorf, Switzerland) and analyzed using Magellan software (TECAN). The raw data was measured as 450nm optical density (OD) and the concentration was calculated using a linear standard curve generated from 6–7 standards. The lower limit of quantification (LLOQ) was determined using the lowest standard. The calculation of the LLOQ was performed by subtracting the OD of the background (diluent) from the OD of the lowest standard concentration and back calculating the concentration from the standard curve.

**2D Echocardiography.** 24 hours after CLP, naïve mice (n=5) or ertapenem-treated CLP mice (n=10) were anesthetized with isoflurane and their left ventricle (LV) was imaged by echocardiography using a high-resolution imaging system (Vevo 770, Visual Sonics, Canada). LV internal distances, heart rate, and posterior wall thickness were measured for the calculation of various parameters of LV structure and function. LV volume and ejection fraction (EF) were calculated using the Teichholz formula^29^, and related parameters were calculated as previously described^30^.

**Bioenergetics analysis.** *Cell isolation, seeding, and analysis*. 24 hours after CLP, mice were euthanized, the spleen was extracted and splenocytes were dissociated. cells were seeded at a density of 0.5×10^6^ cells/well into XF96 well plates pre-coated with poly-D-lysine (100 μg/mL) to maximize adherence and allowed to adhere overnight. After recording of basal measurements, the Mito Stress Test (Agilent, Santa Clara, CA, USA) injection strategy consisted of oligomycin (1 μM), FCCP (1 μM), and rotenone/antimycin A in combination (1 μM). The Glycolytic Stress Test (Agilent) injection strategy consisted of glucose (10 mM) and oligomycin (1 μM), followed by 50 mM 2-deoxyglucose (2DG). Oxygen consumption rate (OCR) and extracellular acidification rate (ECAR) were measured with the XF96 Extracellular Flux Analyzer (Seahorse Bioscience, North Billerica, MA, USA) using three 3 min cycles of mix and measurement following each injection. *Normalization:* Upon completion of the extracellular flux assay, plated cells were lysed, and their protein concentrations were quantified using the BCA assay. Briefly, cells were lysed with 50 μl RIPA lysis medium supplemented with protease inhibitors for each well and agitated for 5 min, cells were incubated at RT for 30 min, and post-incubation lysate samples were added to BCA working reagent medium and measured for absorbance at 562 μm. *Data analysis:* Assay data were analyzed with MS Excel, using the XF Report Generator macro-enabled spreadsheet (Agilent).

***Statistics.*** Differences between groups were examined for statistical significance using the Mann-Whitney nonparametric test. Differences between multiple groups were examined for statistical significance using Kruskal–Wallis one-way analysis of variance (non-parametric ANOVA) with multiple-comparisons adjusted by using the Dunn’s test. Lung/body weight ratio was examined using the one-way analysis of variance (ANOVA). Correlation of any parameter to clinical score was evaluated by a Spearman's rank correlation coefficient, with a coefficient higher than 0.7 or lower than -0.7 being a strong correlation. All statistical analyses were done using GraphPad Prism (San Diego, CA, USA). Survival analysis was performed according to the Kaplan-Meier method. A Log-rank statistical test was performed using GraphPad Prism.

***Study approval.*** The experimental procedures for the animal studies were approved by the Institutional Animal Care and Use Committee of the Hebrew University Medical School.

**Supplementary References**

1. Marshall, J. C. *et al.* Multiple organ dysfunction score: a reliable descriptor of a complex clinical outcome. *Crit. Care Med.* **23**, 1638–52 (1995).

2. Vincent, J.-L. Organ Dysfunction in Patients with Severe Sepsis. *Surg. Infect. (Larchmt).* **7**, s-69-s-72 (2006).

3. Peters, E., Masereeuw, R. & Pickkers, P. The potential of alkaline phosphatase as a treatment for sepsis-associated acute kidney injury. *Nephron - Clin. Pract.* **127**, 144–148 (2014).

4. Pettengill, M. *et al.* Human alkaline phosphatase dephosphorylates microbial products and is elevated in preterm neonates with a history of late-onset sepsis. *PLoS One* **12**, e0175936 (2017).

5. Fawley, J. & Gourlay, D. M. Intestinal alkaline phosphatase: a summary of its role in clinical disease. *J. Surg. Res.* **202**, 225–234 (2016).

6. Bauerle, J. D., Grenz, A., Kim, J. H., Lee, H. T. & Eltzschig, H. K. Adenosine generation and signaling during acute kidney injury. *J. Am. Soc. Nephrol.* **22**, 14–20 (2011).

7. Zimmermann, H., Zebisch, M. & Sträter, N. Cellular function and molecular structure of ecto-nucleotidases. *Purinergic Signal.* **8**, 437–502 (2012).

8. Davidson, J. A. *et al.* Alkaline Phosphatase, Soluble Extracellular Adenine Nucleotides, and Adenosine Production after Infant Cardiopulmonary Bypass. *PLoS One* **11**, e0158981 (2016).

9. Davidson, J. A. *et al.* Alkaline Phosphatase Activity and Endotoxemia After Infant Cardiothoracic Surgery. *Shock* **51**, 328–336 (2019).

10. Koyama, I., Matsunaga, T., Harada, T., Hokari, S. & Komoda, T. Alkaline phosphatases reduce toxicity of lipopolysaccharides in vivo and in vitro through dephosphorylation. *Clin. Biochem.* **35**, 455–61 (2002).

11. Coletta, C. *et al.* Endothelial dysfunction is a potential contributor to multiple organ failure and mortality in aged mice subjected to septic shock: preclinical studies in a murine model of cecal ligation and puncture. *Crit. Care* **18**, 511 (2014).

12. Qin, S. *et al.* Role of HMGB1 in apoptosis-mediated sepsis lethality. *J. Exp. Med.* **203**, 1637–42 (2006).

13. Charchaflieh, J., Rushbrook, J., Worah, S. & Zhang, M. Activated Complement Factors as Disease Markers for Sepsis. *Dis. Markers* **2015**, 382463 (2015).

14. Charchaflieh, J. *et al.* The Role of Complement System in Septic Shock. *Clin. Dev. Immunol.* **2012**, 1–8 (2012).

15. Flierl, M. A. *et al.* Functions of the complement components C3 and C5 during sepsis. *FASEB J.* **22**, 3483–3490 (2008).

16. Ward, P. A. Sepsis, apoptosis and complement. *Biochem. Pharmacol.* **76**, 1383–8 (2008).

17. Younger, J. G. *et al.* Complement activation in emergency department patients with severe sepsis. *Acad. Emerg. Med.* **17**, 353–9 (2010).

18. Stöve, S. *et al.* Circulating complement proteins in patients with sepsis or systemic inflammatory response syndrome. *Clin. Diagn. Lab. Immunol.* **3**, 175–83 (1996).

19. Voll, R. E. *et al.* Immunosuppressive effects of apoptotic cells. *Nature* **390**, 350–1 (1997).

20. Unnewehr, H. *et al.* Changes and regulation of the C5a receptor on neutrophils during septic shock in humans. *J. Immunol.* **190**, 4215–25 (2013).

21. Ward, P. A. The dark side of C5a in sepsis. *Nat. Rev. Immunol.* **4**, 133–142 (2004).

22. Hartemink, K. J. & Groeneveld, A. B. J. The hemodynamics of human septic shock relate to circulating innate immunity factors. *Immunol. Invest.* **39**, 849–862 (2010).

23. Ren, J. *et al.* Complement Depletion Deteriorates Clinical Outcomes of Severe Abdominal Sepsis: A Conspirator of Infection and Coagulopathy in Crime? *PLoS One* **7**, 1–9 (2012).

24. Yuan, Y., Yan, D., Han, G., Gu, G. & Ren, J. Complement C3 depletion links to the expansion of regulatory T cells and compromises T-cell immunity in human abdominal sepsis: A prospective pilot study. *J. Crit. Care* **28**, 1032–1038 (2013).

25. Hollmann, T. J., Mueller-Ortiz, S. L., Braun, M. C. & Wetsel, R. A. Disruption of the C5a receptor gene increases resistance to acute Gram-negative bacteremia and endotoxic shock: opposing roles of C3a and C5a. *Mol. Immunol.* **45**, 1907–15 (2008).

26. Trahtemberg, U. & Mevorach, D. Apoptotic Cells Induced Signaling for Immune Homeostasis in Macrophages and Dendritic Cells. *Front. Immunol.* **8**, 1356 (2017).

27. Ren, Y. *et al.* Apoptotic Cells Protect Mice against Lipopolysaccharide-Induced Shock. *J. Immunol.* **180**, 4978–85 (2008).

28. Morioka, S. *et al.* Efferocytosis induces a novel SLC program to promote glucose uptake and lactate release. *Nature* **563**, 714–718 (2018).

29. Teichholz, L. E., Kreulen, T., Herman, M. V. & Gorlin, R. Problems in echocardiographic volume determinations: Echocardiographic-angiographic correlations in the presence or absence of asynergy. *Am. J. Cardiol.* **37**, 7–11 (1976).

30. Stypmann, J. *et al.* Echocardiographic assessment of global left ventricular function in mice. *Lab. Anim.* **43**, 127–137 (2009).

**Supplementary Table 1.** 2D Echocardiography parameter analysis

| **Parameter** | **Median of Naïve [IQR]** | | **Median of CLP [IQR]** | | **^2^P- Value** | **^3^Correlation to Clinical Score** |
| --- | --- | --- | --- | --- | --- | --- |
| **^1^**Heart rate (BPM); HR | 500 [458, 561]; | N=5 | 358.5 [270, 409]; | N=10 | 0.003 | - 0.878 |
| **^4^**Fractional shortening (%); FS | 30 [28.45, 40.8]; | N=5 | 40.4 [30.6, 51.6]; | N=9 | n.s | No |
| **^5^**Ejection fraction (%); EF | 57.7 [55.3, 71.45]; | N=5 | 71.9 [59.1, 84.15]; | N=9 | n.s | No |
| Posterior wall thickness (mm); PWT | 25 [19.55, 37.65]; | N=5 | 15 [5.3, 50.25]; | N=9 | n.s | No |
| **^6^**LV Volume- Diastole (µl); LVEDV | 68.3 [56.8, 82.7]; | N=5 | 39.1 [26.9, 50.05]; | N=9 | 0.0035 | - 0.701 |
| **^6^**LV Volume- Systole (µl); LVESV | 29 [17.35, 37.05]; | N=5 | 11.8 [4.2, 17.25]; | N=9 | 0.018 | - 0.597 |
| LV Area- Diastole (mm^2^); LVEDA | 10.3 [9.4, 11.58]; | N=5 | 8.44 [5.95, 8.72]; | N=9 | 0.002 | No |
| LV Area- Systole (mm^2^); LVESA | 5.15 [4.62, 5.94]; | N=5 | 3.57 [2.09, 4.76]; | N=9 | 0.042 | No |
| **^7^**Fractional area shortening (%); FAS | 54.42 [26.32, 58.36]; | N=5 | 54.54 [40.37, 70.93]; | N=9 | n.s | No |
| **^8^**LV Stroke volume (µl); SV | 41.3 [38.5, 45.65]; | N=5 | 23.4 [21.25, 37.5]; | N=9 | 0.007 | - 0.691 |
| **^9,1^**Cardiac output (ml/min); CO | 20.62 [18.41, 24.6]; | N=5 | 9.34 [7.33, 11.92]; | N=9 | 0.002 | - 0.799 |

LV internal distances (diastole/systole, LVIDD, and LVIDS, respectively), HR and PWT were measured in duplicates or triplicates using the M-Mode view of the echocardiograms; LVEDA and LVESA were measured using the B-Mode view of the echocardiograms.

**^1^**Significant difference between CLP mice and naïve mice with a strong correlation to MSS Clinical Score (-0.7 > ρ- Spearman > 0.7)

**^2^** Mann-Whitney 2-tailed nonparametric t-test; **^3^** ρ- Spearman; **^4^** $FS (\%)=\frac{LVIDD-LVIDS}{LVIDd}\times100$; **^5^** $EF (\%)=\frac{LVEDV-LVESV}{LVEDV}\times100$

**^6^** Teichholz method for LV volume calculation: *LVEDV (µl)*$=\frac{7 \times{LVIDD}^{3}}{[2.4+LVIDD]}$; *LVESV (µl)*$=\frac{7 \times{LVIDS}^{3}}{[2.4+LVIDS]}$; **^7^** $FAS (\%)=\frac{LVEDA-LVESA}{LVEDA}\times100$;

**^8^** $SV \left( \mu l \right)=LVEDV-LVESV$; **^9^** $CO \left( \frac{ml}{min} \right)=SV\times HR$ / 1000

**Supplementary Table 2.** Mitochondrial function and glycolysis assay analysis of mice derived splenocytes, 24h post-CLP (N= number mice)

| **^1^Assay** | **Parameter** | **Median of Naïve [IQR]** | | **Median of CLP [IQR]** | | **^2^P- Value** | **^3^Correlation to Clinical Score** |
| --- | --- | --- | --- | --- | --- | --- | --- |
| **Mitochondrial Respiration (OCR)** | Non-mitochondrial oxygen consumption | 4.92 [4.45, 4.79]; | N=6 | 3.89 [3.64, 4.43]; | N=18 | 0.0071 | -0.7191 |
|  | Basal respiration | 14.63 [12.58, 16.08]; | N=6 | 13.13 [10.78, 14.08]; | N=18 | 0.0769 | -0.5253 |
|  | Maximal respiration | 42.46 [30.56, 45.83]; | N=6 | 28.9 [25.0, 33.53]; | N=18 | 0.0224 | -0.4288 |
|  | Proton leak | 3.19 [2.16, 3.6]; | N=6 | 3.52 [3.14, 4.23]; | N=18 | 0.1556 | No |
|  | ATP production | 11.37 [10.48, 12.26]; | N=6 | 9.57 [7.11, 10.18]; | N=18 | 0.0010 | -0.7547 |
|  | Spare respiratory capacity | 27.18 [18.27, 30.71]; | N=6 | 17.37 [13.54, 20.94]; | N=18 | 0.0180 | No |
|  | Spare respiratory capacity as a % of Basal respiration | 278 [237, 309]; | N=6 | 236 [206, 267]; | N=18 | 0.1994 | No |
|  | Coupling efficiency (%) | 79 [75.7, 82.5]; | N=6 | 70 [68.7, 73.2]; | N=18 | 0.0001 | -0.7902 |
| **Glycolytic function (ECAR)** | Non-glycolytic acidification | 0.94 [0.81, 1.1]; | N=6 | 0.89 [0.77, 0.95]; | N=17 | 0.3452 | No |
|  | Glycolytic capacity | 0.67 [0.49, 0.84]; | N=6 | 0.74 [0.6, 0.85]; | N=17 | 0.6447 | No |
|  | Glycolysis | 1.24 [1.08, 1.72]; | N=6 | 1.18 [0.93, 1.34]; | N=17 | 0.3262 | No |
|  | Glycolytic reserve | 0.69 [0.50, 0.88]; | N=6 | 0.42 [0.21, 0.51]; | N=17 | 0.0149 | -0.4993 |
|  | Glycolytic reserve as a % of Glycolysis | 205 [188, 241]; | N=6 | 151 [133, 173]; | N=17 | 0.0063 | -0.4875 |

**^1^**Measurements were carried out in triplicate and are normalized to the cell concentration, as determined by BCA protein quantification assay. **^2^** Mann-Whitney 2-tailed nonparametric t-test; **^3^** ρ- Spearman

**Supplementary Table 3.** 24-hour serum cytokine/chemokine change in mice after CLP-induced sepsis (N=6) compared to naïve mice (N=2)

| **Analyte** | **CLP compared to naïve mice** |
| --- | --- |
| CRP | ↑↑ |
| ENA-78 | ↑↑ |
| Eotaxin | ↑↑ |
| G-CSF | ↑↑↑ |
| GM-CSF | ↑↑ |
| Gro-α | ↑↑ |
| IL-1α | ↑↑ |
| IL-1β | ↑↑↑ |
| IL-2 | ↑↑ |
| IL-2R | ↑↑ |
| IL-5 | ↑↑ |
| IL-6 | ↑↑↑ |
| IL-9 | ↑↑ |
| IL-10 | ↑↑↑ |
| IL-12p70 | ↑↑ |
| IL-17A | ↑↑↑ |
| IL-18 | ↑↑ |
| IL-22 | ↑↑↑ |
| IL-23 | ↑↑↑ |
| IL-27 | ↑↑↑ |
| IL-28 | ↑↑ |
| IL-31 | ↑↑ |
| IFNγ | ↑↑ |
| IP-10 | ↑↑↑ |
| LIF | ↑↑↑ |
| MCP-1 | ↑↑ |
| MCP-3 | ↑↑ |
| MIP-1α | ↑↑ |
| MIP-1β | ↑↑ |
| MIP-2 | ↑↑↑ |
| RANTES | ↑↑↑ |
| TNFα | ↑↑ |
| VEGF-A | ↑↑ |
