# Supplementary material for: Apoptotic cell therapy for cytokine storm associated with acute severe sepsis
Source: Cell Death Dis. 2020 Jul 15;11(7):535. doi: 10.1038/s41419-020-02748-8 (PMC7363887; doi:10.1038/s41419-020-02748-8)
Supplement: Supplementary file 2 — Supplementary Figure Legends [file 41419_2020_2748_MOESM2_ESM.docx]

**Supplementary Figure Legends**

**Content:**

**Supplementary Fig. 1 legend**

**Supplementary Fig.1. Representative analysis of AnnexinV and PI for Allocetra-OTS cells injected to mice.** 250,000 Allocetra-OTS cells were incubated for 10min with AnV-FITC an PI on ice (MBL, MA, USA) in 1.5mM calcium buffer. Cells were analyzed by FACSCalibur (BD, NJ, USA) and FCS express software (DeNovo, CA, USA). (**a**) Cells were gated based on their size and granularity; (**b**) Gated cells were then analyzed for AnnexinV/PI staining.
